# Remineralization of dentin slices using casein phosphopeptide–amorphous calcium phosphate combined with sodium tripolyphosphate

**DOI:** 10.1186/s12938-020-0756-9

**Published:** 2020-04-03

**Authors:** Zhou Zhou, Xingyun Ge, Minxia Bian, Tao Xu, Na Li, Jiamin Lu, Jinhua Yu

**Affiliations:** 1 Institute of Stomatology, Nanjing Medical University, 136 Hanzhong Road, Nanjing, 210029 Jiangsu China; 2 Key Laboratory of Oral Diseases of Jiangsu Province and Stomatological Institute of Nanjing Medical University, 140 Hanzhong Road, Nanjing, 210029 Jiangsu China; 3 Endodontic Department, School of Stomatology, Nanjing Medical University, 136 Hanzhong Road, Nanjing, 210029 Jiangsu China

**Keywords:** CPP–ACP, TPP, Demineralized dentin slices, Remineralization

## Abstract

**Background:**

The remineralization approach mechanically occludes the exposed dentinal tubules mechanically, reduces the permeability of dentinal tubules and eliminates the symptoms of dentin hypersensitivity. The aim of the present study was to investigate the remineralization of demineralized dentin slices using CPP–ACP combined with TPP, and the research hypothesis was that CPP–ACP combined with TPP could result in extrafibrillar and intrafibrillar remineralization of dentin.

**Methods:**

Demineralized dentin slices were prepared and randomly divided into the following groups: A (the CPP–ACP group), B (the CPP–ACP + TPP combination group), C (the artificial saliva group), D (the negative control group), and E (the positive control group). Dentin slice samples from groups A, B and C were remineralized and the remineralization effect was evaluated using scanning electron microscopy (SEM), transmission electron microscopy (TEM), energy-dispersive X-ray spectroscopy (EDX), attenuated total reflection–Fourier transform infrared spectroscopy (ATR–FTIR) and X-ray diffraction (XRD).

**Results:**

Treatment with CPP–ACP combined with TPP occluded the dentinal tubules and resulted in remineralization of collagen fibrils. The hydroxyapatite crystals formed via remineralization were found to closely resemble the natural dentin components.

**Conclusion:**

CPP–ACP combined with TPP has a good remineralization effect on demineralized dentin slices.

## Background

Dentin hypersensitivity is a commonly found symptom in stomatology and it is usually caused by loss of enamel and demineralization of dentin leading to symptoms of sore teeth following dentin irritation [1]. The worldwide prevalence of dentin hypersensitivity ranges from 8 to 74% worldwide [2–4]. Dentin hypersensitivity can be treated with nerve stabilization or with potassium and dental laser irradiation [5]. Chemical inhibition or alteration of the nerve impulse using potassium nitrate reduces the sensitivity of teeth. Nevertheless, this strategy irritates the pulp and the effect is unclear. In addition, the use of dental lasers is still not common in general dental clinical practice. The most robust strategy to reduce dentine hypersensitivity entails painless and non-invasive remineralization, leading to the formation of a remineralized layer on dentin. The remineralization approach mechanically occludes the exposed dentinal tubules mechanically, reduces the permeability of dentinal tubules and eliminates the symptoms of dentin hypersensitivity [6, 7].

Dentin is a mineralized collagenous tissue and demineralized dentin does not significantly induce the deposition of calcium phosphate minerals in the remineralization solution. This is because there is no residual crystal on the surface of the demineralized dentin, which is detrimental to the deposition of calcium phosphate minerals. This top-down mineralization method does not depend on the spontaneous nucleation of minerals on organic substrates but on the epitaxial growth of residual apatite seeds [8, 9]; thus, only loosely stacked plate crystals are present. In contrast, bottom-up methods assemble materials at nanoscale to form larger structures. The ideal approach to dentin remineralization is the “bottom-up” non-classical method. It is different from classical remineralization, which depends on pre-existing apatite crystals, but it is a biomimetic method mimicking the natural biological conditions [10]. Biomimetic non-collagen analogs and molecules are generally used for remineralization of dentin. The commonly used biomimetic molecules include casein phosphopeptide–amorphous calcium phosphate (CPP–ACP), bioglass, polyacrylic acid (PAA), synthetic polypeptide and polyamide-amine dendrimer (PAMAM) [11–14].

CPP–ACP has been shown to significantly promote remineralization of enamel subsurface lesions and reduce progression of approximal caries, and it has been successfully incorporated into oral health products [15]. CPP–ACP is formed by the complexation of casein phosphopeptide (CPP) with amorphous calcium phosphate (ACP) via a phosphorylated peptide chain (containing sequence SPSPSPEE) [16]. Since CPP has a space compartment effect, which prevents the formation and growth of calcium phosphate crystals, calcium phosphate is probably complexed with CPP in an amorphous state to form a stable CPP–ACP nanocomposite [17, 18]. CPP–ACP readily translocates calcium and phosphate ions into the demineralization zone of the tooth with a concentration gradient, which inhibits demineralization and promotes remineralization of teeth. CPP–ACP has been used for remineralization of enamel in the past. Unfortunately, CPP–ACP is rarely used for remineralization of dentin, as intrafibrillar remineralization of dentin using CPP–ACP is challenging [19, 20]. To date, there have been few reports on the ability of intrafibrillar remineralization of dentin. Recent studies have indicated that sodium tripolyphosphate (TPP) may be used as a biomimetic analog of dentin matrix protein 1 (DMP1) and as a non-collagen protein in dentin to induce specific binding to collagen microfibrils [21, 22]. Although TPP has been frequently employed as an additive in the food industry, it can also phosphorylate dentin collagen [23]. In this study, TPP was used to phosphorylate dentin collagen fibrils to increase collagen surface crystallization and induce collagen mineralization. In addition, the ACP was stabilized by the CPP–ACP complex to promote TPP-labeled crystallization for complete remineralization.

The aim of the present study was to investigate the remineralization of demineralized dentin slices using CPP–ACP combined with TPP, and the research hypothesis was that CPP–ACP combined with TPP could result in extrafibrillar and intrafibrillar remineralization of dentin.

## Results

### Analysis of micromorphological changes

**Fig. 1:**
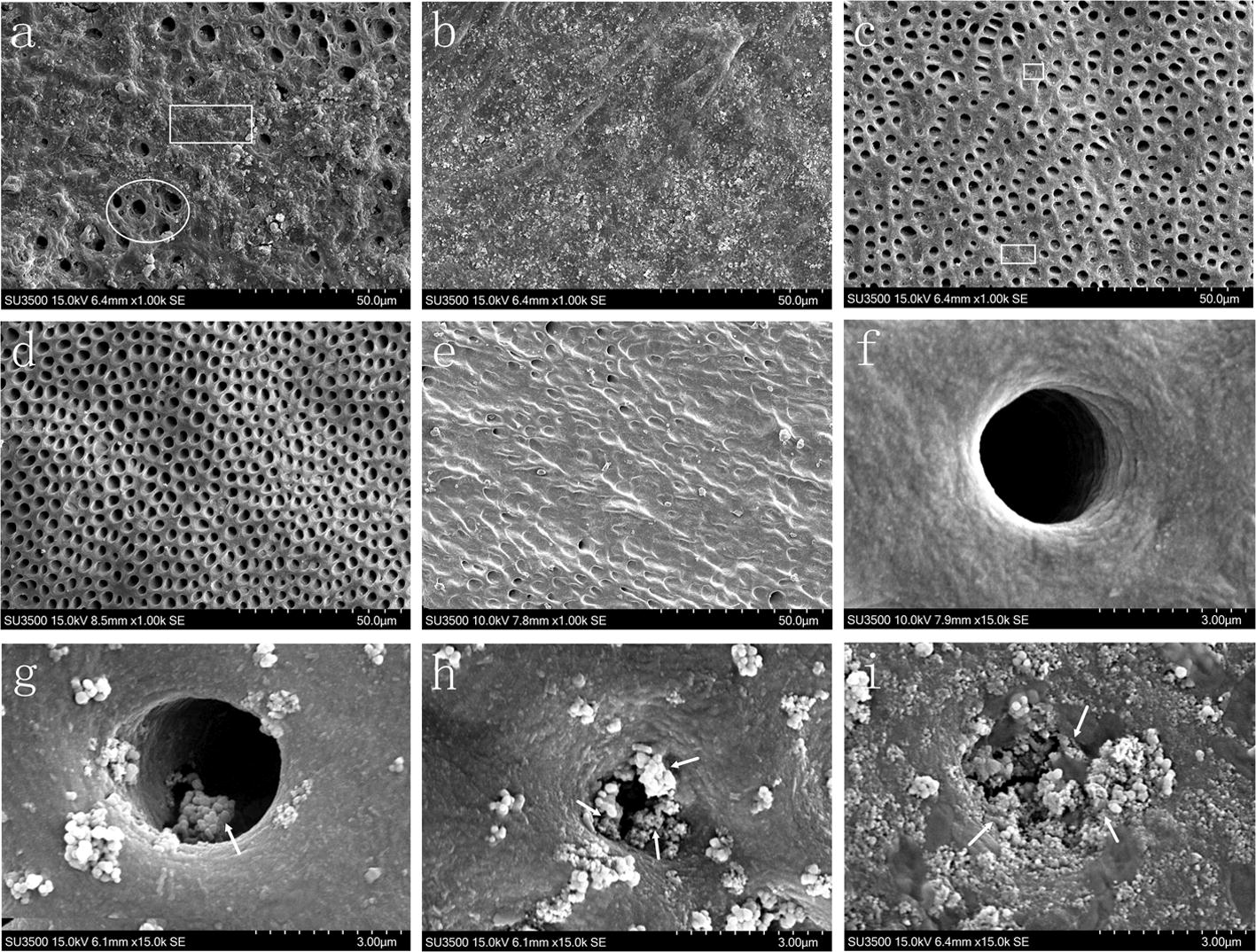
Scanning electron microscopy (SEM) micrographs of dentin slices. **a** SEM image (×1000) of Group A after treatment for 21 days; **b** SEM image (×1000) of Group B after treatment for 21 days; **c** SEM image (×1000) of Group C after treatment for 21 days; **d** SEM image (×1000) of a demineralized dentin slice in Group D; **e** SEM image (×1000) of a natural dentin slice in Group E; **f** SEM image of a demineralized dentin slice (×15,000); **g** SEM image (×15,000) of Group B after treatment for 7 days; **h** SEM image (×15,000) of Group B after treatment for 14 days; (i) SEM image (×15,000) of Group B after treatment for 21 days. The rectangular area represents the occluded area of dentin tubules, the circular area is the unoccluded area of dentin tubules, and the area indicated by the arrow is the remineralized material

The five groups of dentin slice samples were analyzed via SEM after dehydration, gold spray and conductive adhesive fixation on the workbench. The results showed that the surface deposits of groups A and B gradually increased over time. After 21 days, the surface of the dentin slices in group B was generally covered by a new mineralization layer, and the dentinal tubules were well occluded (Fig. 1b). The surface dentin in group A also contained large mineral deposits, but the sediment was not homogeneous. In addition, a handful of dentinal tubules were unoccluded by the new minerals in group A (Fig. 1a). In group C, only a few minerals were deposited after 21 days and the dentinal tubules were not substantially occluded by remineralization (Fig. 1c). The dentinal tubules on the surface of the demineralized dentin slice samples from group D, the negative control group, were mostly open (Fig. 1d); whereas the surface of the dentin samples without demineralization in group E, the positive control group, remained closed (Fig. 1e). SEM results obtained at a high magnification (15,000×) showed that the dentinal tubules in group B were gradually occluded by remineralization over time, and were completely occluded after 21 days (Fig. 1f–i).

**Fig. 2:**
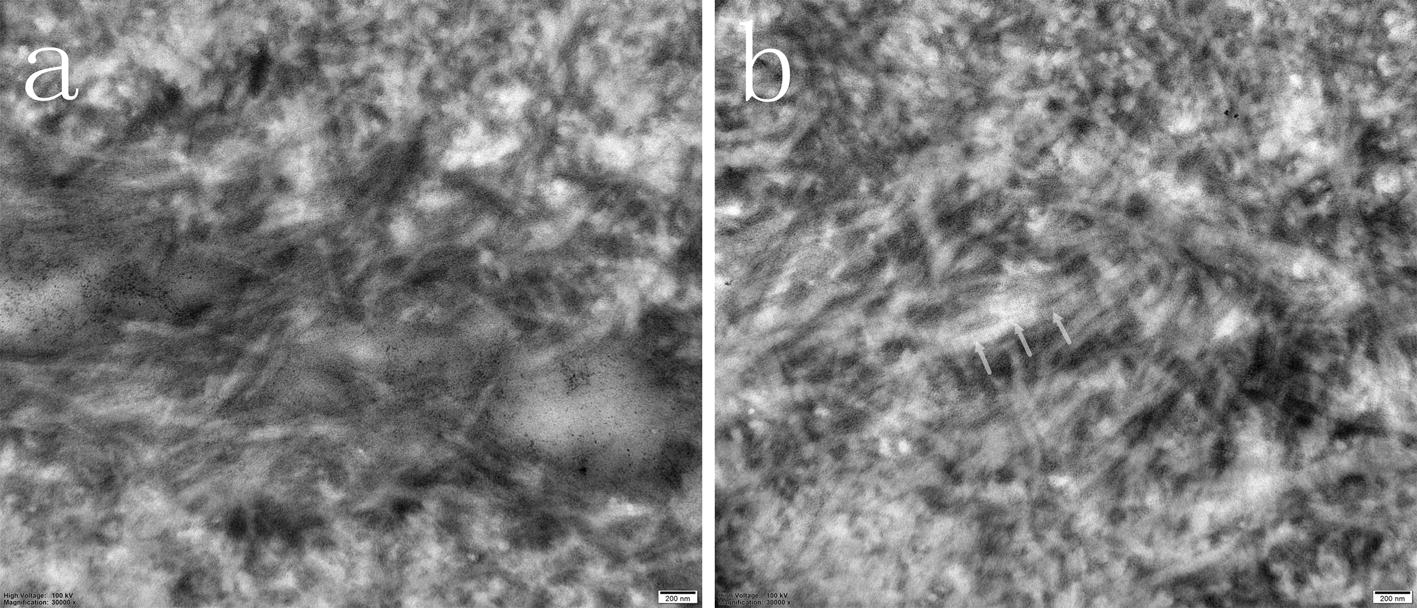
Transmission electron microscopy (TEM) micrographs of remineralized dentin slices. **a** TEM image (×30,000) of Group A after treatment for 21 days; **b** TEM image (×30,000) of Group B after treatment for 21 days. The periodic transverse bands were found in the dentin collagen fibrils of group B (arrow)

After 21 days of treatment, the TEM results did not reveal a periodic transverse band structure in the dentin collagen fibrils of group A (Fig. 2a); whereas the dentin collagen fibrils in group B presented periodic transverse bands (Fig. 2b). The periodic transverse band structure is the periodically arranged intrafibrillar apatite platelet, which indicates interfibrillar mineralization.

### Quantitative analysis of changes in calcium (Ca) and phosphorus (P) compositions composition of remineralized dentins via EDX

**Fig. 3:**
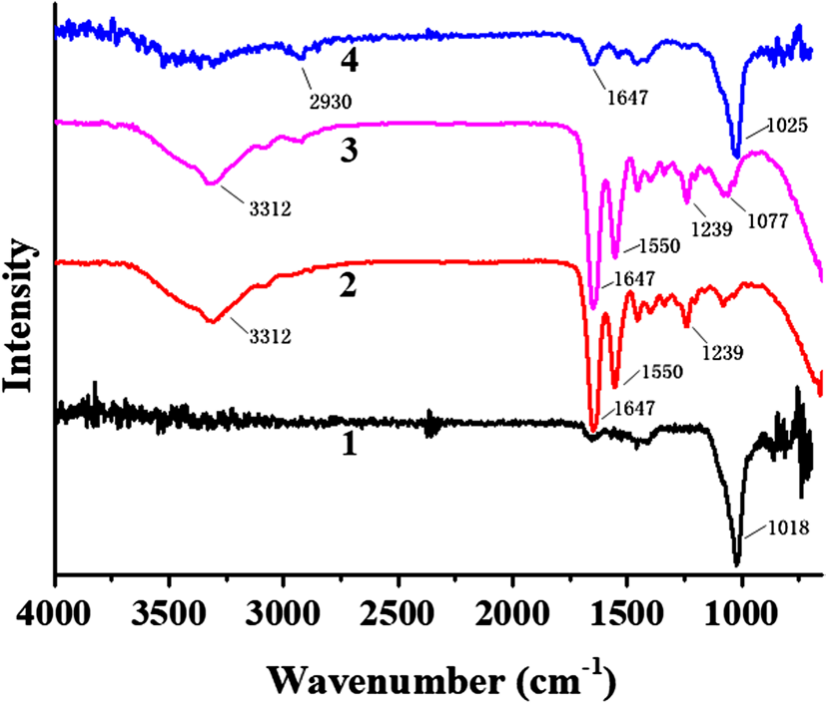
ATR–FTIR spectra of dentin slices surface. Spectrum 1, a natural dentin slice; Spectrum 2, a demineralized dentin slice; Spectrum 3, a demineralized dentin slice after TPP treatment; Spectrum 4, a demineralized dentin slice after remineralization with CPP–ACP combined with TPP for 21 days. The peak value at 1018 cm^−1^ indicated the anti-symmetric stretching vibration peak of PO4^3−^. The peak at 1647 cm^−1^ was the characteristic peak of amide I band, and 1550 cm^−1^ was the characteristic peak of amide II band. The characteristic peak at 1239 cm^−1^ was amide III band

Hydroxyapatite is the major component required for remineralization. The remineralization process is accompanied by an increase in the ratio of calcium to phosphorus, which is an important indicator of the remineralization effect [13]. Elemental analysis of the sample surface in each group was conducted using EDX. The calcium/phosphorus molar ratios for mineralization on the surface of the dentin slices are presented in Table 1 and Fig. 3. The calcium/phosphorus (Ca/P) molar ratio in group B was close to the sample value in the positive control group E, and there was no statistically significant difference (*P *> 0.05). Additionally, the Ca/P molar ratio in group B was significantly higher than that in group C and group D (*P* < 0.05); while the Ca/P molar ratio in group A was only significantly higher than that in group D (*P* < 0.05), indicating the satisfactory remineralization of the samples in group B, and that the quality of the hydroxyapatite crystal formed was close to that of natural dentin.

**Table 1:** Ca/P ratios of each group

Group	Number of observations	Mean ± SD
Group A (CPPACP)	5	1.58 ± 0.05^bc^
Group B (TPP + CPPACP)	5	1.61 ± 0.04^c^
Group C (artificial saliva)	5	1.52 ± 0.03^ab^
Group D (negative)	5	1.49 ± 0.04^a^
Group E (positive)	5	1.64 ± 0.03^c^

The difference between Group B and Group E is not statistically significant (*P *> 0.05), while differences between Group B and Group C, between Group B and Group D and between Group A and Group D are statistically significant (*P *< 0.05)
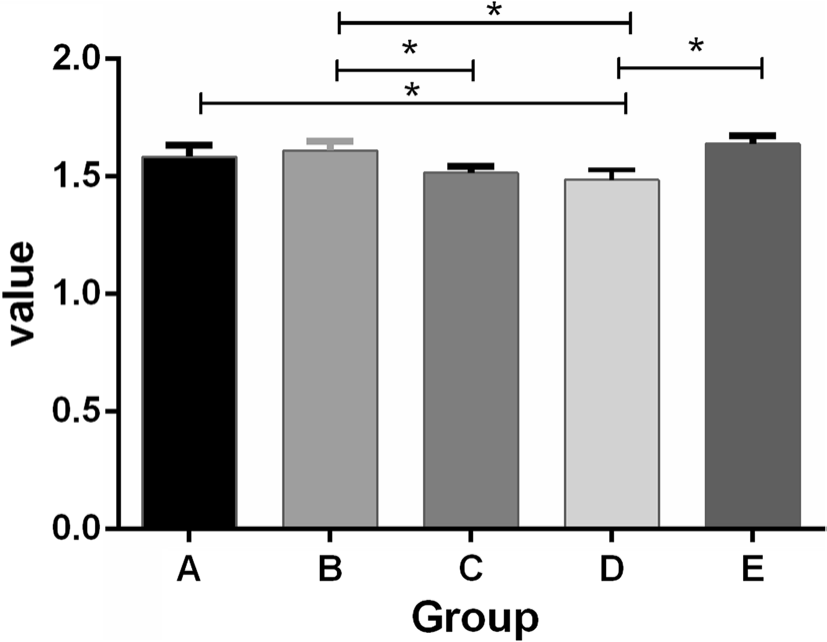

* *P* < 0.05

Comparasion of Ca/P ratios in each group. The difference between Group B and Group E is not statistically significant (*P *> 0.05), while differences between Group B and Group C, between Group B and Group D and between Group A and Group D are statistically significant (*P *< 0.05)

^a–c^There was no statistical significant difference between the groups marked with the same footnote

### Functional analysis of each sample by ATR–FTIR

The results of ATR–FTIR are presented in Fig. 3. Spectrum 1 represents a sample in the positive control group, a natural dentin sample; Spectrum 2 refers to a sample in the negative control group, the demineralized dentin sample; Spectrum 3 is the result of a demineralized dentin sample after TPP treatment; and Spectrum 4 represents a sample of demineralized dentin after remineralization with CPP–ACP combined with TPP for 21 days. In natural dentin slices, the main component was hydroxyapatite crystals, and the visible wave number was 1018 cm^−1^ and the anti-symmetrical stretching peak of P–O bond was located at the peak of PO4^3−^. After the dentin slices were demineralized, the peak appeared at 3312 cm^−1^, which was the stretching vibration peak of –OH bond. The characteristic peak at 1647 cm^−1^ was the amide I band, the one at 1550 cm^−1^ was the characteristic peak of amide II band, the one at 1239 cm^−1^ was the characteristic peak of amide III band, and the peaks at 1374 cm^−1^ and 1465 cm^−1^ were the bending vibration peaks of CH. The peak indicated that the dentin slices had been demineralized. After demineralized dentin slices were phosphorylated with TPP, the intensity of the peak at 1077 cm^−1^ was significantly enhanced, which indicated that the demineralized dentin slices had completed phosphorylated. After the demineralized dentin samples were remineralized by TPP and CPP–ACP for 21 days, a weak peak at 2930 cm^−1^ was the stretching vibration peak of CH, a weak characteristic peak of the amide band appeared, and the peak at 1025 cm^−1^ was the characteristic absorption peak of PO4^3−^, indicating that the remineralization of dentin had been completed. The infrared spectrum of demineralized dentin slices showed that they had undergone remineralization by use of combined TPP and CPP–ACP for 21 days. The peak positions and peak intensities of the infrared spectrum of the essential samples were roughly the same as those of the natural dentin slices, which proved that a large number of hydroxyapatite crystals were deposited on the surface.

### Changes in the crystal structure

**Fig. 4:**
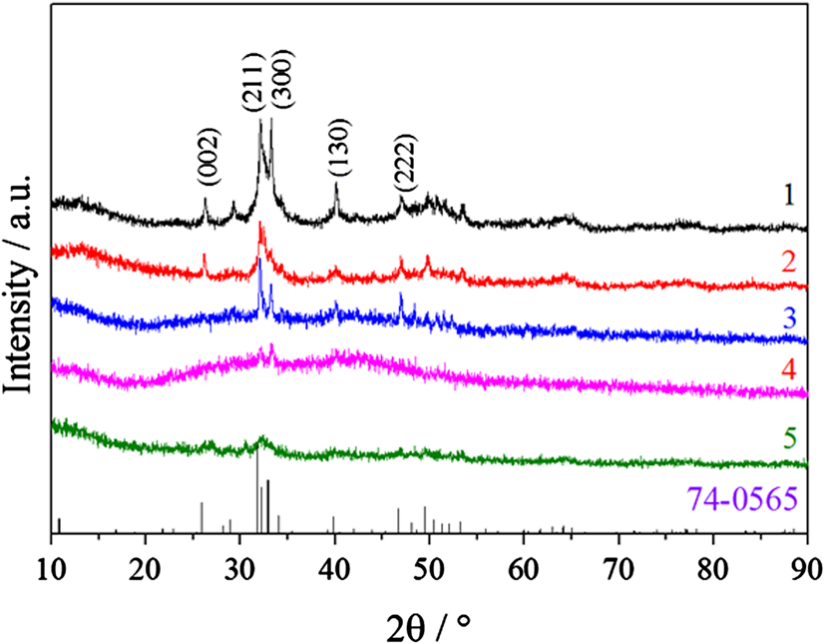
XRD spectra of dentin slices surface. Spectrum 1, a natural dentin slice; Spectrum 2, a remineralized dentin slice following treatment with CPP–ACP combined with TPP for 21 days; Spectrum 3, a remineralized dentin slice following treatment just with CPP–ACP for 21 days; Spectrum 4, a remineralized dentin slice following treatment with only artificial saliva; Spectrum 5, a demineralized dentin slice. The PDF card 74-0565 showed the standard diffraction peak pattern of Ca_10_(PO_4_)_6_(OH)_2_. After 21 days of combined remineralization with TPP and CPP–ACP, the bulge almost completely disappeared at 25º–45º, and the (002) peak appeared, which was very close to the natural dentin diffraction pattern, indicating that most of the amorphous hydroxyapatite had transformed into a crystalline state

The surface structure of the dentin slices was characterized by XRD (Fig. 4). Spectrum 1 represents the peak pattern of the natural dentin; Spectrum 2 shows the peaks of remineralized dentin following treatment with CPP–ACP combined with TPP for 21 days; Spectrum 3 is the peak spectrum of remineralized dentin following treatment with CPP–ACP for 21 days; Spectrum 4 is the result of remineralized dentin with only artificial saliva; and Spectrum 5 is the peak spectrum of the demineralized dentin slices. The PDF card 74-0565 showed the standard diffraction peak pattern of Ca_10_(PO_4_)_6_(OH)_2_. The XRD results of demineralized dentin slices showed that they only bulged at around 32º, indicating that there was no crystalline hydroxyapatite; thus, complete demineralization was achieved. After remineralization with artificial saliva for 21 days alone, the (211) and (300) peaks of hydroxyapatite appeared, which meant that crystalline hydroxyapatite began to appear but only in a small amount, resulting in no other diffraction peaks. The bulge at 25º–45º indicated a large amount of amorphous hydroxyapatite. The 21-day curve of CPP–ACP alone showed that remineralization was suppressed at 25º–45º, and the peaks of (211) and (300) were enhanced and the peaks of (130) and (222) appeared. The amorphous hydroxyapatite was reduced and the hydroxyapatite content was increased. After 21 days of combined remineralization with TPP and CPP–ACP, the bulge almost completely disappeared at 25º–45º, and the (002) peak appeared, which was very close to the natural dentin diffraction pattern, thus indicating that most of the amorphous hydroxyapatite had transformed into a crystalline state, and the demineralized dentin slices showed substantial remineralization.

## Discussion

Remineralization of dentin is more difficult than that of enamel, mainly due to structural differences. Enamel is mainly composed of inorganic hydroxyapatite materials. After demineralization of the enamel, the residual apatite crystals continuously attract calcium and phosphorus ions for epitaxial deposition and remineralization. In contrast, dentin mainly consists of organic components including collagen fibrils. Slight demineralization can be reversed via remineralization of the surface, and growth and reproduction of the crystal cell. However, when the dentin is dramatically demineralized, remineralization via the traditional approach of residual crystal deposition is not feasible [24].

Collagen fibrils are the main organic component of dentin, which provide a scaffold for mineral deposition during remineralization. However, the collagen fibril cannot control the mineral deposition and the remineralization process mainly depends on non-collagen proteins in dentin [6]. The natural process of dentin mineralization occurs via regulation of non-collagen protein in conjunction with deposition of calcium and phosphorus elements on dentin collagen fibrils, followed by formation of hydroxyapatite to complete the mineralization process [25].

Biomimetic remineralization simulates the regulatory process of the non-collagen component in dentin by specific compounds and it is accomplished using calcium and phosphorus ions. CPP is a bioactive peptide extracted from casein in milk by trypsinization and shows excellent biocompatibility [26–28]. CPP–ACP also binds to the surface of *Streptococcus* mutants to inhibit their adhesion, which inhibits bacterial proliferation, kills bacteria, and prevents caries [29]. CPP–ACP is a non-collagen analog that has been used widely in enamel remineralization due to its biosafety [18, 19], but it is less frequently used in dentin remineralization. It is difficult to achieve intrafibrillar remineralization of dentin using CPP–ACP alone [24].

Biomimetic remineralization of dentin entails not only the deposition of minerals on the surface of collagen fibrils in dentin, but also more importantly, their entry into the collagen fibrils for complete mineralization within the fibrils [24, 29]. TPP is an amorphous water-soluble linear polyphosphate with good biocompatibility. In this study, the dentin collagen fibrils were first phosphorylated by TPP to provide nucleation sites for the entry of calcium and phosphorus ions, and the precursors stabilized by CPP–ACP penetrated the collagen fibrils to complete the intrafibrillar remineralization process. The orientation of the crystals was first perpendicular to the long axis of the fibril, and it gradually aligned parallel to the long axis of the fibril, resulting in a distinct transverse band structure. This process showed complete self-assembly and alignment of hydroxyapatite crystals in the dentin collagen fibrils [30].

The results of microscopic observation of dentin morphology showed that group A (CPP–ACP) and group B (TPP + CPP–ACP) showed a large degree of remineralization on the surface of the dentin treated for 21 days, and the dentinal tubules were generally occluded. The remineralization in group B was more even than that in group A. The TEM results showed that the periodic transverse bands in the dentin collagen fibrils appeared in group B after remineralization for 21 days, but not in group A, thus suggesting that CPP–ACP combined with TPP resulted in intrafibrillar remineralization in group B; whereas, CPP–ACP remineralization alone led to poor intrafibrillar remineralization. The quantitative analysis of Ca and P elements in remineralization by EDX showed that the ratio of calcium to phosphorus in group B was greater than that in group A and was close to that in the natural dentin. The elemental composition results confirmed that dentin remineralization in group B yielded better results. Meanwhile, from the perspective of functional groups and crystal structure components, TPP combined with CPP–ACP achieved a better remineralization effect. In the field of remineralization of dentin or enamel, usually only the ratio of calcium and phosphorus is calculated as an indicator of remineralization; and EDX can meet this need. The same sample could be directly subjected to EDX detection after SEM detection, which maintained the uniformity and continuity of the sample. Another advantage of EDX in the study was that it could be performed without damaging the sample. Therefore, in most of the research on remineralization of dentin or enamel, EDX is still used to detect the ratio of calcium and phosphorus [13, 31, 32].

Treatment with CPP–ACP alone led to remineralization of the demineralized dentin slices over time, but the mineral deposition was disordered. The use of TPP in conjunction with CPP–ACP for the treatment of demineralized dentin slices enhanced the remineralization effect compared with CPP–ACP alone. Such a combination resulted in intrafibrillar remineralization of dentin and restored the mechanical properties of dentin slices after remineralization [24, 33]. TPP and CPP–ACP may be used as biomimetic molecules to simulate the N- and C-termini of the non-collagen substances in dentin, respectively. After the crystal nucleation site was formed, the amorphous calcium phosphate precursor was stabilized to the corresponding position to achieve intrafibrillar and interfibrillar collagen remineralization, which mimicked the self-assembly and alignment of mineral crystals during natural mineralization. The current rate of biomimetic remineralization is relatively low, thus suggesting the need for more efficient strategies and options for minimally invasive dental treatment.

## Conclusion

Treatment with CPP–ACP combined with TPP results in extrafibrillar and intrafibrillar remineralization of dentin, and it is superior compared to CPP–ACP alone.

## Methods

### Preparation of demineralized dentin slice samples

The study was approved by the ethics committee of the Affiliated Stomatological Hospital of Nanjing Medical University, and the patients’ written informed consent was obtained. The patients’ third molar teeth were completely extracted in the outpatient oral surgery department. The teeth were disinfected with 0.005% thymol solution and stored at 4 °C. The surface enamel of the dentin was removed and a low-speed diamond saw (IsoMet Low Speed Saw, Buehler, Lake Bluff, IL, US) was utilized to obtain 55 slice samples measuring 4 × 4 × 1 mm in size. The slice specimens were sequentially polished with 180 grit, 400 grit, 800 grit and 1200 grit carbide polishing papers and transferred into an ultrasonic cleaner containing deionized water and washed for 5 min to remove the smear layer. Five slices served as the untreated positive control group. The remaining dentin slices were submerged in 20% ethylenediaminetetraacetic acid (EDTA) solution for 2 weeks to simulate dentine demineralization. The demineralized dentin slices were rinsed in the ultrasonic cleaner with deionized water and dried for further use.

### The dentin slice samples were randomly divided into groups A, B and C containing 15 samples each, and groups D and E containing 5 samples each

1. Group A: The CPP–ACP (Tooth Mousse, GC International, Itabashi-ku, Tokyo, Japan) was evenly applied to the surface layers of the demineralized dentin slices for 3 min and the samples were rinsed with deionized water. The processed dentin slices were transferred to a 15-mL test tube containing artificial saliva (1.5 mmol/L CaCl_2_, 0.9 mmol/L KH_2_PO_4_, 20 mmol/L Hepes, 150 mmol/L NaCl, pH 7.0) [34], and incubated in a water bath at a constant temperature of 37 °C. The CPP–ACP was applied twice every 24 h and the artificial saliva was replaced each time. After 7 days, 14 days and 21 days, the dentin slice samples were removed and rinsed with deionized water in an ultrasonic cleaning machine for 2 min and dried.
2. Group B: The demineralized dentin slice samples were pre-treated with a solution of 25 g/L TPP for 24 h, washed and dried, followed by coating with CPP–ACP gel for 3 min. The rest of the treatment steps were similar to those for group A.
3. Group C: The demineralized dentin slice samples were placed in a 15-mL test tube containing artificial saliva. The test tube was incubated in a constant temperature water bath at 37 °C. The artificial saliva was replaced every 24 h. After 7, 14, and 21 days, 5 remineralized dentin slice samples were retrieved, washed in deionized water with an ultrasonic cleaner and dried.
4. Group D: The negative control group contained untreated samples of demineralized dentin slices.
5. Group E: The positive control group comprised samples of natural dentin slices without acid etching or demineralization.

### Remineralization of the demineralized dentin slices

The dentin samples obtained from each group were imaged using scanning electron microscopy (SEM) (Hitachi-SU3500, Hitachi, Ltd., Tokyo, Japan) with a beam voltage of 15 kV to observe the surface morphology and evaluate the occlusion status and integrity of dentinal tubules. Transmission electron microscopy (TEM) (Tecnai Spirit 120kv, FEI, Hillsboro, OR, US) was used to determine the “transverse band” structure, a typical intrafibrillar remineralization marker, of the collagen fibrils in the remineralized dentin.

Energy-dispersive X-ray spectroscopy (EDX) (AZtecEnergy, Oxford Instruments, Oxfordshire, UK) was used to identify the changes in Ca and P composition of hydroxyapatite on the surface of the remineralized dentin slices. Samples in each group were scored at 5 random spots for analysis, and the data obtained were analyzed using one-way ANOVA. The analysis was performed using SPSS 13.0, and *P*-values less than 0.05 were considered statistically significant.

The changes in organic composition of the dentin slices were assessed via attenuated total reflection–Fourier transform infrared spectroscopy (ATR–FTIR) (NEXUS670, Nicolet Instrument Co, Madison, WI, US). The working parameters of ATR–FTIR were as follows: scanning range: 700–4000 cm^−1^; resolution: 4 cm^−1^; and scanning frequency: 32.

The surface crystalline components were analyzed by X-ray diffraction (XRD) (D8 Advance, Bruker axs GmbH, Karlsruhe, Germany). The working parameters of XRD were as follows: Cu target radiation (wavelength: l.54056 × 10^−10^ m); tube current: 40 mA; tube voltage: 40 kV; scanning speed: 6° min^−1^ (each step: 0.02°, 0.2 s/step); and scanning range: 10°–80°.

**Fig. 5:**
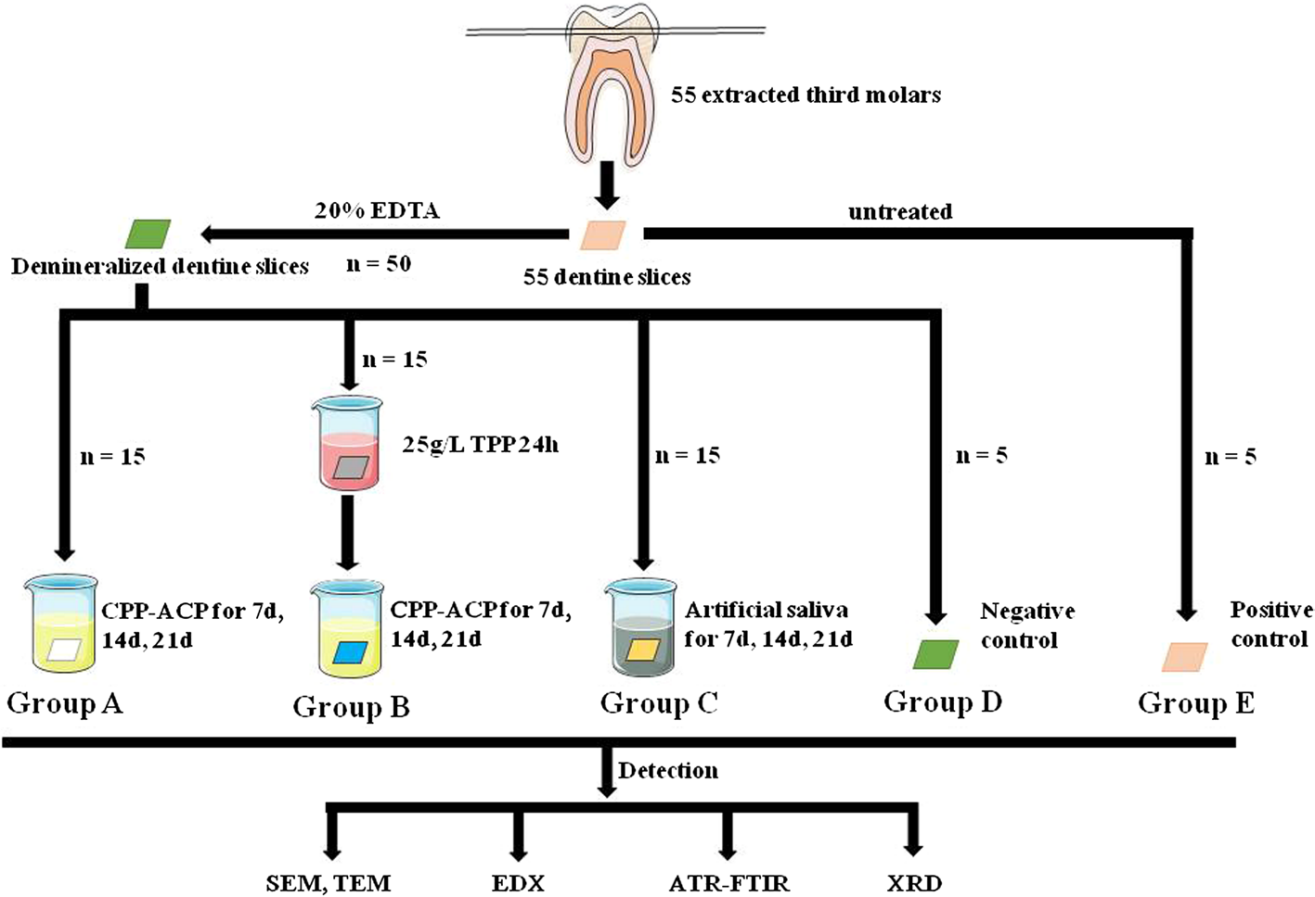
Schematic representation of the design and procedures of the study

In this manner, the remineralization of demineralized dentin slices was analyzed based on changes in surface morphology, internal structure and mineral composition. The design and procedures of this study are summarized in Fig. 5.

## Data Availability

The datasets analyzed during the current study can be made available upon request.

## Abbreviations

CPP–ACP: Casein phosphopeptide–amorphous calcium phosphate
TPP: Sodium tripolyphosphate
PAA: Polyacrylic acid
PAMAM: Polyamide-amine dendrimer
CPP: Casein phosphopeptide
ACP: Amorphous calcium phosphate
DMP1: Dentin matrix protein 1
EDTA: Ethylenediaminetetraacetic acid
SEM: Scanning electron microscopy
TEM: Transmission electron microscopy
EDX: Energy dispersive X-ray spectroscopy
ATR–FTIR: Attenuated total reflection–Fourier transform infrared spectroscopy
XRD: X-ray diffraction
